# Special Issue: The Role of Complement in Cancer Immunotherapy

**DOI:** 10.3390/antib10030029

**Published:** 2021-07-23

**Authors:** Ronald P. Taylor

**Affiliations:** Department of Biochemistry and Molecular Genetics, University of Virginia School of Medicine, Charlottesvile, VA 22908, USA; rpt@virginia.edu; Tel.: +1-434-987-1964

The complement system plays an important role in critical aspects of immune defense and in the maintenance of homeostasis in the bloodstream, as well as in essentially all tissues and organs [1]. More than 100 years ago, Bordet was awarded the Nobel Prize for his discovery of complement. His work revealed that antibodies complexed with antigens activate complement and induce substantial inflammation, leading to cell and tissue destruction, and therefore it is not surprising that numerous clinical and basic science investigations have focused on “The Role of Complement in Cancer Immunotherapy”, which is the topic of this Special Issue. Within the context of this title, the 10 articles in this issue examine a wide range of subjects, and this range illustrates the diverse and at times contradictory actions of complement in cancer.

The development of mAb technologies and the application of mAbs in cancer immunotherapy has led to a continuing and exponential phase of investigation and initiation of clinical trials, first punctuated by FDA approval of CD20 mAb rituximab for the treatment of B-cell lymphomas [2,3]. Although the efficacy of rituximab was and is clearly demonstrable, its apparent mechanisms of action were the subject of considerable controversy; however, its putative “apoptotic induction” has been set aside, and its therapeutic action has been clearly demonstrated to require immune effector mechanisms, which include complement-dependent cytotoxicity (CDC) [2,3]. Moreover, the basic science studies of rituximab have led to findings that have been most immediately applicable to understanding how other tumor-specific mAbs function, and the outcome of the studies of rituximab have also set the stage for the development of much more effective second- and third-generation mAbs that target CD20 as well as other tumor-associated antigens [4].

In terms of maintaining homeostasis, complement promotes wound healing and angiogenesis (essential to cell growth) and there is a substantial literature that describes how complement can establish an environment that allows for growth of tumors. This is most evident when the tumors are *not* recognized as foreign, and therefore can take advantage of the “cell growth”-promoting action of complement [1,5]. The encyclopedic review of Revel and colleagues comprehensively describes complement pathways as well as the numerous cases in which specific complement components, especially C1q and C5a, play important roles both in promoting tumor growth and in generating an immunosuppressive environment [1]. Weak immune responses to the tumors (titers of IgG and IgM insufficient to mediate cell killing) appear to activate and recruit complement proteins to foster cell growth, and the complement components can be produced by the host or generated by the tumors themselves. Revel et al. also document in mouse models the roles that complement can play in suppressing or promoting tumor growth; this again illustrates the apparent and unresolved contradiction between “promotor or suppressor of cancer progression”.

Thurman and colleagues also recognize the “dysfunctional relationship” between complement and cancer, and review their interesting and provocative findings which have demonstrated that inflammation associated with complement activation can induce downstream oxidative damage and transformation (but not killing) of cells which leads to malignancy [6]. They note that non-lethal complement activation within the tumor microenvironment (TME) also promotes angiogenesis, thereby providing a favorable niche for a growing tumor. Moreover, they review a voluminous literature documenting the increased expression of complement control proteins on cancer cells, which is clearly an additional defensive measure that cancer cells appear to have evolved to avoid potential lethal cytotoxic “side effects” of modest complement activation. They also cite the seminal studies of Markiewski and Lambris, who first demonstrated that the C5a produced by cancer cells can attract myeloid-derived suppressor cells (MDSC) to the TME, thus providing yet another “pro-tumor” defensive activity of complement.

The review by Markiewski and colleagues focuses on complement-mediated (neo) angiogenesis, which helps to provide a blood supply to the growing tumor [5]. They report that complement influences the generation of the “Premetastatic Niche” in which, due to the action of C5a, MDSC are recruited before the arrival of tumor cells. These processes are described in exquisite detail, and the authors make clear that the factors mediating angiogenesis for tumors are also operative in other pathologies, including age-related macular degeneration (AMD). They note that targeting of certain complement factors including C5aR1 may be effective in the treatment of both cancer and AMD.

The role of complement and, in particular, complement receptors in mouse models of triple-negative breast cancer are examined in two particularly interesting and innovative articles [7,8]. This form of cancer lacks any of the common and targetable hormone receptors and is therefore particularly resistant to most conventional treatments. The groups of Woodruff and Rolfe examined the influence of the well-studied agent PMX53 (C5aR1 *antagonist*) as well as an *agonist* for both C3aR and C5aR1 (EP54), on the growth of syngeneic mammary carcinoma cell lines in mice [7]. The investigators report that in contrast to findings in several other mouse cancer models, PMX53 had no effect on tumor growth in this system. In most other models, the PMX53 suppressed tumor growth by interfering with “recruitment” of MDSC to the TME. On the other hand, while the EP54 agonist suppressed tumor growth, its actual mechanism of action was not clear, but was most likely due to enhancement of T-lymphocyte action in the TME. These findings once again emphasize the complex role of complement in cancer, and indicate that directed therapies based on complement must be carefully and specifically designed; it appears that there are few general rules that apply.

Teams led by Peerschke and Ghebrehiwet have been among the leading groups studying the biology and immunology of C1q and its receptors. In this issue, they have investigated how targeting the globular receptor to C1q (gC1qR) with a specific neutralizing mAb affects the growth of breast cancer cells in a mouse xenotranplant model of triple-negative breast cancer in which the gC1qR is upregulated on the tumor cells [8]. The investigators made use of a variety of elegant immunostaining techniques along with measures of tumor growth and report, for the first time, in vivo proof of principle for suppression of growth of triple-negative breast cancer cells accomplished by targeting gC1qR with a neutralizing mAb. Mechanisms of action of the mAb may include both induction of apoptosis of the tumor cells as well as inhibition of angiogenesis. Additional studies of these phenomena and possible translation to the clinic are anticipated.

Many of the key studies of the mechanisms of CD20 mAb-mediated killing of primary tumor cells (in most cases malignant B cells from patients with chronic lymphocytic leukemia (CLL)) have been reported by groups led by Golay or Zent [2,3]. The review by Golay and Taylor highlights the ex vivo studies in whole blood that were pioneered by Golay and Introna [3]. This work has clearly established that upon binding to CLL B cells, both rituximab and ofatumumab (but not obituzumab) make use of complement which most immediately kills B cells, and both groups have demonstrated that B cells opsonized with complement fragments can also be eliminated by immune cells that express receptors for both IgG (Fc receptors) and C3 fragments (such as CR3) in a synergistic process. Although these reactions are quite effective, they can be overwhelmed at high B-cell burdens, thus leading to exhaustion of complement, both in vivo and in vitro. Whether use of fresh frozen plasma as a complement source can effectively restore and or enhance the immediate action of these mAbs remains under investigation. Zent and colleagues have made use of a bedside to bench approach and find that phagocytosis of opsonized cells by macrophages appears to be the principal and most important mechanism by which B cells opsonized with rituximab or ofatumaub are eliminated [2]. Their work with primary CLL cells has definitively established that higher concentrations of mAbs (and therefore higher levels of opsonization of target cells) are required for CDC than for phagocytosis. Golay and Taylor also cite several bedside to bench investigations which indicate that upregulation of complement control proteins is *not* a mechanism of resistance employed by CLL cells to inhibit CDC mediated by rituximab or ofatumumab. Finally, both reviews discuss the important advance in the design of more effective complement-activating mAbs based on generating IgG molecules that more readily form hexamers upon binding to cells, thus allowing for more effective chelation of C1q [4]. The illustrations in both of these articles are elegant and informative.

Rosskopf and colleagues have made substantial advances in the area of IgG engineering by modifying mAbs to enhance their potential to more effectively target and destroy tumor cells [9]. They have made use of an FDA-approved CD19 mAb (tafasitamab) which has modest activity in inducing antibody-dependent cellular cytotoxicity (ADCC) but does not activate complement or induce CDC. They recognize that a key issue in engineering such IgG1 mAbs is that the C1q-binding regions and Fc-chelating sites on the mAbs are close together, thus presenting a challenge to generating a mAb with substantial levels of both activities. They found that by making directed changes in the amino acid sequence of the Fc region (EFTAE modification) along with expressing its afucosylated form, they were able to produce a mAb with considerably higher levels of both ADCC and CDC. The experimental methodologies used by the investigators encompass protein engineering, cellular binding assays of mAbs and C1q and functional CDC and ADCC assays, and are all elegant, rigorous and very well described. Their approaches provide a template for additional efforts in the optimization of mAbs for cancer immunotherapy.

The review by Taylor and Lindorfer examines in detail key steps in complement mediated lysis of B cells that are opsonized with highly effective complement-activating mAbs (hexamer-forming) specific for CD20 and CD37 [4]. There is indeed considerable co-localization of cell-bound mAb with C1q, and this is rapidly followed by “nearby” covalent deposition of C3b (colocalized with bound mAb), which is soon followed by assembly and deposition of the membrane attack complex (MAC, C5b-9) of complement. Ultimately the most direct cause of cell death is due to influx of lethal amounts of Ca^2+^ mediated by the MAC, and the entire process is complete in just a few minutes. One surprising outcome of these studies was the observation that CLL cells (not a cell line!) could indeed be killed by this mechanism *in the absence of C9,* thereby revealing that the Ca^2+^ influx mediated by the smaller C5b-8 pore was adequate to rapidly kill the cells. These observations can therefore set the standard for the generation and testing of future tumor-specific mAbs that make use of complement for cancer immunotherapy. It is most likely that as other highly effective complement fixing mAbs are developed, many of the phenomena described by Taylor and Lindorfer will be closely replicated with other cancer cells and their cognate mAbs. Whether CDC can be accomplished in the absence of C9 for these yet-to-be-developed mAbs is not clear, but based on the observations with the traditionally resistant CLL cells it is likely that C9 *will not* be required. Another lesson learned from these studies is that the action of mAbs that are particularly effective at activating complement exceeds the molecular thresholds for C1q binding and C3b deposition required for the generation of large quantities of the MAC, and therefore these mAbs are capable of overwhelming the natural defenses (including complement control proteins) expressed by tumor cells [4].

Elvington, Liszewski and Atkinson have written a comprehensive review on the complement control protein CD46, membrane cofactor protein (MCP), which was first described by the Atkinson lab more than 30 years ago [10]. Most research on this molecule has focused on human systems because CD46 is not expressed in mice. The protein is present on a wide variety of human cells and in addition to its many immunologic functions, it has been demonstrated to be the cell entry site of several viruses, including adenovirus and measles virus. Moreover, there is considerable evidence that CD46 is substantially overexpressed on tumor cells, and therefore it is under intense investigation as a prime target for cancer immunotherapy. These investigations include the use of CD46-directed mAb–drug conjugates; the CD46 mAb is specific for a conformational epitope expressed *only* on cancer cells. Other therapies under investigation make use of modified forms of measles virus or adenovirus that are engineered to only replicate in cancer cells (oncolytic viruses). As noted by the authors, the CD46 target is now the subject of more than 20 clinical trials in a variety of cancers, including melanoma and pancreatic cancer. Early on, it was discovered that CD46 protects cells from complement attack by serving as a co-factor for inactivation of deposited C3b and C4b. Therefore, it may be particularly interesting to examine the CDC activity against tumor cells by the cancer cell-specific CD46 mAb after it is engineered to form hexamers upon binding to cells. By specifically binding at high levels, only to CD46 expressed on the cancer cells, the hexamer-forming mAb may be quite effective at exceeding the molecular threshold for C3b deposition (see above) required for mediating downstream complement activation and cell lysis.

The paradoxical effects of the complement system in cancer will continue to present challenges in the development of effective mAbs for cancer immunotherapy. The 10 articles featured in this Special Issue should therefore be of considerable interest to basic scientists and to physician scientists investigating new approaches in the immunotherapy of cancer.
